# Non-isovalent substitution in a Zintl phase with the TiNiSi type structure, CaMg_1–*x*_Ag_*x*_Ge [*x* = 0.13 (3)]

**DOI:** 10.1107/S1600536809048454

**Published:** 2009-11-21

**Authors:** Charles Banenzoué, Siméon Ponou, John Ngolui Lambi

**Affiliations:** a Departement de Chimie Inorganique, Université de Douala, Cameroon; b Department of Inorganic Chemistry, Arrhenius Laboratory, Stockholm University, SE 106 91 Stockholm, Sweden; c Department of Chemistry, E.N.S. de Yaounde, BP 47 Yaounde, Cameroon

## Abstract

Single crystals of the title Ag-substituted calcium magnesium germanide, CaMg_1–*x*_Ag_*x*_Ge [*x* = 0.13 (3)] were obtained from the reaction of the corresponding elements at high temperature. The compound crystallizes with the TiNiSi structure type (Pearson code *oP*12) and represents an Ag-substituted derivative of the Zintl phase CaMgGe in which a small fraction of the divalent Mg atoms have been replaced by monovalent Ag atoms. All three atoms in the asymmetric unit (Ca, Mg/Ag, Ge) occupy special positions with the same site symmetry (.*m*.). Although the end member CaAgGe has been reported in an isomorphic superstructure of the same TiNiSi type, higher Ag content in solid solutions could not be achieved due to competitive formation of other, perhaps more stable, phases.

## Related literature

For the KHg_2_ structure type, see: Duwell & Baenziger (1955 ▶) and for the TiNiSi structure type, see: Shoemaker & Shoemaker (1965 ▶); Eisenmann *et al.* (1972 ▶); Villars & Calvert (1991 ▶). For the structural systematics and properties of the TiNiSi structure type, see: Kauzlarich (1996 ▶); Landrum *et al.* (1998 ▶). For related compounds, see: Ponou & Lidin (2008 ▶); Ponou *et al.* (2007 ▶). For atomic radii, see: Pauling (1960 ▶).

## Experimental

### 

#### Crystal data

CaMg_0.87_Ag_0.13_Ge
*M* *_r_* = 147.84
Orthorhombic, 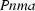

*a* = 7.5128 (2) Å
*b* = 4.4573 (1) Å
*c* = 8.3911 (2) Å
*V* = 280.99 (1) Å^3^
*Z* = 4
Mo *K*α radiation
μ = 13.43 mm^−1^
*T* = 293 K
0.10 × 0.06 × 0.04 mm

#### Data collection

Oxford Xcalibur3 diffractometer
Absorption correction: multi-scan (*CrysAlis RED*; Oxford Diffraction, 2007 ▶) *T*_min_ = 0.396, *T*_max_ = 0.584
2462 measured reflections
516 independent reflections
481 reflections with *I* > 2σ(*I*)
*R*_int_ = 0.026

#### Refinement

*R*[*F* ^2^ > 2σ(*F* ^2^)] = 0.019
*wR*(*F* ^2^) = 0.041
*S* = 1.10
516 reflections
21 parameters
Δρ_max_ = 0.89 e Å^−3^
Δρ_min_ = −0.44 e Å^−3^

### 

Data collection: *CrysAlis CCD* (Oxford Diffraction, 2007 ▶); cell refinement: *CrysAlis RED* (Oxford Diffraction, 2007 ▶); data reduction: *CrysAlis RED*; program(s) used to solve structure: *SHELXS97* (Sheldrick, 2008 ▶); program(s) used to refine structure: *SHELXL97* (Sheldrick, 2008 ▶); molecular graphics: *DIAMOND* (Brandenburg, 1999 ▶); software used to prepare material for publication: *SHELXTL* (Sheldrick, 2008 ▶).

## Supplementary Material

Crystal structure: contains datablocks global, I. DOI: 10.1107/S1600536809048454/hb5206sup1.cif

Structure factors: contains datablocks I. DOI: 10.1107/S1600536809048454/hb5206Isup2.hkl

Additional supplementary materials:  crystallographic information; 3D view; checkCIF report

## Figures and Tables

**Fig. 1.**
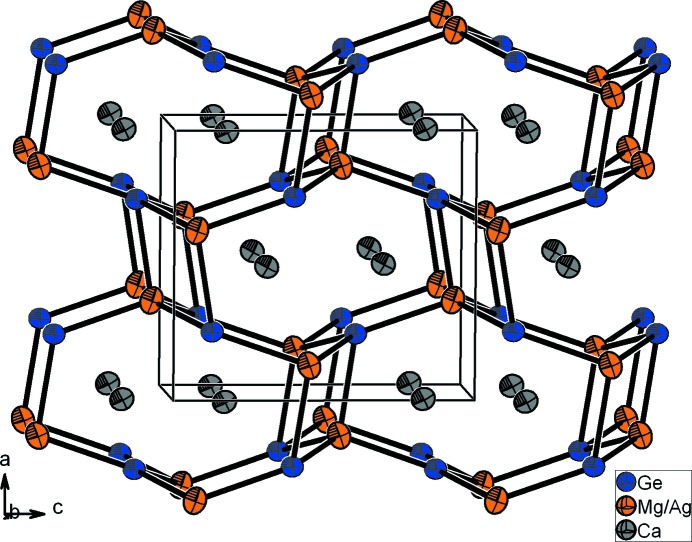
: A perspective view of (I) with displacement ellipsoids drawn at 95% probability level. Ca, Mg/Ag, and Ge atoms are drawn as grey crossed, orange and blue spheres, respectively.

## supplementary crystallographic information

### Comment

The solid solution CaMg_1–_*_x_*Ag*_x_*Ge [*x* = 0.13 (3)] is iso-structural with the non-substituted ternary phase CaMgGe (Eisenmann *et al.*, 1972). It crystallizes in the TiNiSi type (Space Group *Pnma*) with Ca, Mg/Ag, and Ge at Ti, Ni, and Si-positions, respectively. The TiNiSi type (Shoemaker & Shoemaker, 1965) which is well represented among ternary equiatomic phases (Villars & Calvert, 1991), is an ordered ternary derivative of the KHg_2_ type (Duwell & Baenziger, 1955; Pearson code oI12). Hence, the structure consist of a three-dimensional four-connected anionic [Mg_1–_*_x_*Ag*_x_*Ge] networks with the Ca cations sitting in large channels (Fig. 1). The anionic network may be constructed from two-dimensional sheets, similar to those in black phosphorus and running perpendicular to the *a*-axis, which are linked along the *a*-direction to form one-dimensional ladders of edge-sharing four-rings and channels of eight-rings running along the *b*-direction. The TiNiSi type is known to be very versatile and shows remarkable structural and electronic flexibility (Landrum *et al.*, 1998). Meanwhile, a large number of compounds with the TiNiSi type like CaMgGe can be rationalized as Zintl phases (Kauzlarich, 1996) according to the ionic formulation Ca^2+^(Mg^2+^Ge^4–^). Zinlt phases are known to be very sensitive to the electron count (Ponou *et al.*, 2007). But, because of the above mentioned flexibility of the TiNiSi type, non-isovalent substitutions was expected without major structural distortion. Thus, since CaAgGe crystallizes in the isomorphic (*i*_3_) superstructure of the TiNiSi type with a tripling of the *a*-axis (Ponou & Lidin, 2008), a wide stoichiometry breadth was expected in the system CaMg_1–_*_x_*Ag*_x_*Ge. Eventually, the reaction of different starting mixtures with *x* = 1/4, 1/2, and 3/4, yielded almost the same composition (*x* = 0.10 – 0.13) within 3σ standard deviation. This indicates a narrow homogeneity range, meaning that in this class of materials, the inherent electronic rigidity of the Zintl phase may be conflicting with the otherwise remarkable flexibility of TiNiSi type.

CaMg_0.87 (1)_Ag_0.13 (1)_Ge is the Ag-richest phase that was structurally characterized. The unit-cell volume here (V = 280.99 (1) Å^3^) is quite similar to that of the non-substituted phase CaMgGe (V = 280.89 Å^3^), though the size of Mg is significantly larger than [Pauling's (1960) radii: Mg 1.600 Å, Ag 1.440 Å]. But, it should be noted that the later cell parameters were determined with much higher standard deviation (Eisenmann *et al.*, 1972). In a reinvestigation of the CaMgGe structure (not reported), no indications of any superstructure were found.

### Experimental

Three different mixtures of the elements (all from ABCR GmbH, Karlsruhe, Germany) Ca (granule, 99.5%), Mg (pieces, 99.9%), Ag (60*m* powder, 99.9%), and Ge (50*m* powder, 99.999%), with compositions along the CaMg_1–x_Ag*_x_*Ge series with *x* = 1/4, 1/2, and 0.75 were loaded in a Niobium ampoules (approx. 9 mm diameter and 30 mm length) which were sealed on both ends by arc-melting and, in turn, enclosed in evacuated fused silica Schlenk tube to protect the former from air oxidation at high temperature. The ampoules were heated at 1273 K for 2 h, and cooled at a rate of 6 K/h to 923 K, where they are annealed for 24 h, then cooled down to room temperature by turning off the furnace. Semi quantitative EDX analysis of the single crystals confirmed the presenced of the four elements, and no eventual contaminant at the detection limit could be observed.

### Refinement

The refinement was straightforward, the full occupancies for all sites were verified by freeing the site occupation factor for an individual atom, while keeping that of the other atoms fixed. This proved that all positions but one, the Mg site, were fully occupied. The refined occupancy at Mg assigned position was higher than 100%, indicating a mixing with heavier element. Therefore, this position (labelled Mg/Ag) was modelled as a statistical mixture of Mg and Ag, and refined as 86.9 (1)% Mg and 13.1 (1)% Ag.

### Crystal data

**Table:**

CaMg_0.87_Ag_0.13_Ge	*F*(000) = 273.5
*M**_r_* = 147.84	*D*_x_ = 3.495 Mg m^−^^3^
Orthorhombic, *P**n**m**a*	Mo *K*α radiation, λ = 0.71073 Å
Hall symbol: -P 2ac 2n	Cell parameters from 2462 reflections
*a* = 7.5128 (2) Å	θ = 4.9–32.1°
*b* = 4.4573 (1) Å	µ = 13.43 mm^−^^1^
*c* = 8.3911 (2) Å	*T* = 293 K
*V* = 280.99 (1) Å^3^	Irregular block, grey
*Z* = 4	0.10 × 0.06 × 0.04 mm

### Data collection

**Table:**

Oxford Xcalibur3 diffractometer	516 independent reflections
Radiation source: Enhance (Mo) X-ray Source	481 reflections with *I* > 2σ(*I*)
graphite	*R*_int_ = 0.026
Detector resolution: 16.5467 pixels mm^-1^	θ_max_ = 32.1°, θ_min_ = 4.6°
ω scans	*h* = −8→10
Absorption correction: multi-scan (*CrysAlis RED*; Oxford Diffraction, 2007)	*k* = −5→6
*T*_min_ = 0.396, *T*_max_ = 0.584	*l* = −12→12
2462 measured reflections	

### Refinement

**Table:**

Refinement on *F*^2^	0 restraints
Least-squares matrix: full	*w* = 1/[σ^2^(*F*_o_^2^) + (0.0233*P*)^2^] where *P* = (*F*_o_^2^ + 2*F*_c_^2^)/3
*R*[*F*^2^ > 2σ(*F*^2^)] = 0.019	(Δ/σ)_max_ < 0.001
*wR*(*F*^2^) = 0.041	Δρ_max_ = 0.89 e Å^−^^3^
*S* = 1.10	Δρ_min_ = −0.44 e Å^−^^3^
516 reflections	Extinction correction: *SHELXL97* (Sheldrick, 2008), Fc^*^=kFc[1+0.001xFc^2^λ^3^/sin(2θ)]^-1/4^
21 parameters	Extinction coefficient: 0.084 (3)

### Special details

**Table:**

Experimental. CrysAlis RED, (Oxford Diffraction, 2007) Empirical absorption correction using spherical harmonics, implemented in SCALE3 ABSPACK scaling algorithm.
Geometry. All e.s.d.'s (except the e.s.d. in the dihedral angle between two l.s. planes) are estimated using the full covariance matrix. The cell e.s.d.'s are taken into account individually in the estimation of e.s.d.'s in distances, angles and torsion angles; correlations between e.s.d.'s in cell parameters are only used when they are defined by crystal symmetry. An approximate (isotropic) treatment of cell e.s.d.'s is used for estimating e.s.d.'s involving l.s. planes.
Refinement. Refinement of *F*^2^ against ALL reflections. The weighted *R*-factor *wR* and goodness of fit *S* are based on *F*^2^, conventional *R*-factors *R* are based on *F*, with *F* set to zero for negative *F*^2^. The threshold expression of *F*^2^ > σ(*F*^2^) is used only for calculating *R*-factors(gt) *etc*. and is not relevant to the choice of reflections for refinement. *R*-factors based on *F*^2^ are statistically about twice as large as those based on *F*, and *R*- factors based on ALL data will be even larger.

### Fractional atomic coordinates and isotropic or equivalent isotropic displacement parameters (Å^2^)

**Table:**

	*x*	*y*	*z*	*U*_iso_*/*U*_eq_	Occ. (<1)
Ge1	0.76875 (4)	0.2500	0.38457 (3)	0.00994 (12)	
Ag1	0.14396 (8)	0.2500	0.43738 (7)	0.0140 (2)	0.1309 (15)
Mg1	0.14396 (8)	0.2500	0.43738 (7)	0.0140 (2)	0.8691 (15)
Ca1	0.51982 (7)	0.2500	0.68206 (7)	0.01280 (14)	

### Atomic displacement parameters (Å^2^)

**Table:**

	*U*^11^	*U*^22^	*U*^33^	*U*^12^	*U*^13^	*U*^23^
Ge1	0.00976 (16)	0.00827 (16)	0.01178 (17)	0.000	−0.00007 (9)	0.000
Ag1	0.0172 (4)	0.0124 (3)	0.0124 (3)	0.000	−0.0020 (2)	0.000
Mg1	0.0172 (4)	0.0124 (3)	0.0124 (3)	0.000	−0.0020 (2)	0.000
Ca1	0.0119 (3)	0.0134 (3)	0.0131 (3)	0.000	−0.00085 (18)	0.000

### Geometric parameters (Å, °)

**Table:**

Ge1—Mg1^i^	2.7621 (4)	Ag1—Ag1^viii^	3.2788 (9)
Ge1—Ag1^i^	2.7621 (4)	Ag1—Mg1^ix^	3.2788 (9)
Ge1—Mg1^ii^	2.7621 (4)	Ag1—Ag1^ix^	3.2788 (9)
Ge1—Ag1^ii^	2.7621 (4)	Ag1—Ca1^x^	3.3267 (8)
Ge1—Ag1^iii^	2.8535 (7)	Ag1—Ca1^xi^	3.3273 (6)
Ge1—Mg1^iii^	2.8535 (7)	Ag1—Ca1^xii^	3.3273 (6)
Ge1—Mg1^iv^	2.8596 (7)	Ag1—Ca1	3.4912 (8)
Ge1—Ag1^iv^	2.8596 (7)	Ca1—Ge1^ii^	3.1590 (4)
Ge1—Ca1	3.1191 (6)	Ca1—Ge1^i^	3.1590 (4)
Ge1—Ca1^ii^	3.1590 (4)	Ca1—Ge1^xiii^	3.2214 (4)
Ge1—Ca1^i^	3.1590 (4)	Ca1—Ge1^xiv^	3.2214 (4)
Ge1—Ca1^v^	3.2214 (4)	Ca1—Mg1^xv^	3.3267 (8)
Ag1—Ge1^i^	2.7621 (4)	Ca1—Ag1^xv^	3.3267 (8)
Ag1—Ge1^ii^	2.7621 (4)	Ca1—Mg1^xvi^	3.3273 (6)
Ag1—Ge1^vi^	2.8535 (7)	Ca1—Ag1^xvi^	3.3273 (6)
Ag1—Ge1^vii^	2.8596 (7)	Ca1—Mg1^xvii^	3.3273 (6)
Ag1—Mg1^viii^	3.2788 (9)	Ca1—Ag1^xvii^	3.3273 (6)
			
Mg1^i^—Ge1—Ag1^i^	0.00 (2)	Ge1^i^—Ag1—Ca1^x^	63.086 (14)
Mg1^i^—Ge1—Mg1^ii^	107.58 (2)	Ge1^ii^—Ag1—Ca1^x^	63.086 (14)
Ag1^i^—Ge1—Mg1^ii^	107.58 (2)	Ge1^vi^—Ag1—Ca1^x^	82.653 (19)
Mg1^i^—Ge1—Ag1^ii^	107.58 (2)	Ge1^vii^—Ag1—Ca1^x^	177.14 (3)
Ag1^i^—Ge1—Ag1^ii^	107.58 (2)	Mg1^viii^—Ag1—Ca1^x^	60.49 (2)
Mg1^ii^—Ge1—Ag1^ii^	0.0	Ag1^viii^—Ag1—Ca1^x^	60.49 (2)
Mg1^i^—Ge1—Ag1^iii^	71.425 (16)	Mg1^ix^—Ag1—Ca1^x^	60.49 (2)
Ag1^i^—Ge1—Ag1^iii^	71.425 (16)	Ag1^ix^—Ag1—Ca1^x^	60.49 (2)
Mg1^ii^—Ge1—Ag1^iii^	71.425 (16)	Ge1^i^—Ag1—Ca1^xi^	167.565 (19)
Ag1^ii^—Ge1—Ag1^iii^	71.425 (16)	Ge1^ii^—Ag1—Ca1^xi^	84.010 (9)
Mg1^i^—Ge1—Mg1^iii^	71.425 (16)	Ge1^vi^—Ag1—Ca1^xi^	62.269 (13)
Ag1^i^—Ge1—Mg1^iii^	71.425 (16)	Ge1^vii^—Ag1—Ca1^xi^	60.851 (14)
Mg1^ii^—Ge1—Mg1^iii^	71.425 (16)	Mg1^viii^—Ag1—Ca1^xi^	60.470 (16)
Ag1^ii^—Ge1—Mg1^iii^	71.425 (16)	Ag1^viii^—Ag1—Ca1^xi^	60.470 (16)
Ag1^iii^—Ge1—Mg1^iii^	0.00 (2)	Mg1^ix^—Ag1—Ca1^xi^	114.69 (3)
Mg1^i^—Ge1—Mg1^iv^	126.076 (12)	Ag1^ix^—Ag1—Ca1^xi^	114.69 (3)
Ag1^i^—Ge1—Mg1^iv^	126.076 (12)	Ca1^x^—Ag1—Ca1^xi^	120.958 (16)
Mg1^ii^—Ge1—Mg1^iv^	126.075 (12)	Ge1^i^—Ag1—Ca1^xii^	84.010 (9)
Ag1^ii^—Ge1—Mg1^iv^	126.075 (12)	Ge1^ii^—Ag1—Ca1^xii^	167.565 (19)
Ag1^iii^—Ge1—Mg1^iv^	118.073 (19)	Ge1^vi^—Ag1—Ca1^xii^	62.269 (13)
Mg1^iii^—Ge1—Mg1^iv^	118.073 (19)	Ge1^vii^—Ag1—Ca1^xii^	60.851 (14)
Mg1^i^—Ge1—Ag1^iv^	126.076 (12)	Mg1^viii^—Ag1—Ca1^xii^	114.69 (3)
Ag1^i^—Ge1—Ag1^iv^	126.076 (12)	Ag1^viii^—Ag1—Ca1^xii^	114.69 (3)
Mg1^ii^—Ge1—Ag1^iv^	126.075 (12)	Mg1^ix^—Ag1—Ca1^xii^	60.470 (16)
Ag1^ii^—Ge1—Ag1^iv^	126.075 (12)	Ag1^ix^—Ag1—Ca1^xii^	60.470 (16)
Ag1^iii^—Ge1—Ag1^iv^	118.073 (19)	Ca1^x^—Ag1—Ca1^xii^	120.958 (16)
Mg1^iii^—Ge1—Ag1^iv^	118.073 (19)	Ca1^xi^—Ag1—Ca1^xii^	84.104 (19)
Mg1^iv^—Ge1—Ag1^iv^	0.00 (2)	Ge1^i^—Ag1—Ca1	59.328 (12)
Mg1^i^—Ge1—Ca1	73.110 (14)	Ge1^ii^—Ag1—Ca1	59.328 (12)
Ag1^i^—Ge1—Ca1	73.110 (14)	Ge1^vi^—Ag1—Ca1	152.91 (2)
Mg1^ii^—Ge1—Ca1	73.110 (14)	Ge1^vii^—Ag1—Ca1	106.88 (2)
Ag1^ii^—Ge1—Ca1	73.110 (14)	Mg1^viii^—Ag1—Ca1	110.19 (2)
Ag1^iii^—Ge1—Ca1	117.905 (19)	Ag1^viii^—Ag1—Ca1	110.19 (2)
Mg1^iii^—Ge1—Ca1	117.905 (19)	Mg1^ix^—Ag1—Ca1	110.19 (2)
Mg1^iv^—Ge1—Ca1	124.022 (18)	Ag1^ix^—Ag1—Ca1	110.19 (2)
Ag1^iv^—Ge1—Ca1	124.022 (18)	Ca1^x^—Ag1—Ca1	70.261 (14)
Mg1^i^—Ge1—Ca1^ii^	145.884 (18)	Ca1^xi^—Ag1—Ca1	132.669 (11)
Ag1^i^—Ge1—Ca1^ii^	145.884 (18)	Ca1^xii^—Ag1—Ca1	132.669 (11)
Mg1^ii^—Ge1—Ca1^ii^	71.906 (14)	Ge1—Ca1—Ge1^ii^	105.655 (14)
Ag1^ii^—Ge1—Ca1^ii^	71.906 (14)	Ge1—Ca1—Ge1^i^	105.655 (14)
Ag1^iii^—Ge1—Ca1^ii^	134.864 (8)	Ge1^ii^—Ca1—Ge1^i^	89.738 (15)
Mg1^iii^—Ge1—Ca1^ii^	134.864 (8)	Ge1—Ca1—Ge1^xiii^	97.268 (13)
Mg1^iv^—Ge1—Ca1^ii^	66.910 (14)	Ge1^ii^—Ca1—Ge1^xiii^	156.90 (2)
Ag1^iv^—Ge1—Ca1^ii^	66.910 (14)	Ge1^i^—Ca1—Ge1^xiii^	86.771 (6)
Ca1—Ge1—Ca1^ii^	74.345 (14)	Ge1—Ca1—Ge1^xiv^	97.268 (13)
Mg1^i^—Ge1—Ca1^i^	71.906 (14)	Ge1^ii^—Ca1—Ge1^xiv^	86.771 (6)
Ag1^i^—Ge1—Ca1^i^	71.906 (14)	Ge1^i^—Ca1—Ge1^xiv^	156.90 (2)
Mg1^ii^—Ge1—Ca1^i^	145.884 (18)	Ge1^xiii^—Ca1—Ge1^xiv^	87.548 (14)
Ag1^ii^—Ge1—Ca1^i^	145.884 (18)	Ge1—Ca1—Mg1^xv^	126.88 (2)
Ag1^iii^—Ge1—Ca1^i^	134.864 (8)	Ge1^ii^—Ca1—Mg1^xv^	111.240 (16)
Mg1^iii^—Ge1—Ca1^i^	134.864 (8)	Ge1^i^—Ca1—Mg1^xv^	111.240 (16)
Mg1^iv^—Ge1—Ca1^i^	66.910 (14)	Ge1^xiii^—Ca1—Mg1^xv^	49.866 (10)
Ag1^iv^—Ge1—Ca1^i^	66.910 (14)	Ge1^xiv^—Ca1—Mg1^xv^	49.866 (10)
Ca1—Ge1—Ca1^i^	74.345 (14)	Ge1—Ca1—Ag1^xv^	126.88 (2)
Ca1^ii^—Ge1—Ca1^i^	89.738 (15)	Ge1^ii^—Ca1—Ag1^xv^	111.240 (16)
Mg1^i^—Ge1—Ca1^v^	136.591 (17)	Ge1^i^—Ca1—Ag1^xv^	111.240 (16)
Ag1^i^—Ge1—Ca1^v^	136.591 (17)	Ge1^xiii^—Ca1—Ag1^xv^	49.866 (10)
Mg1^ii^—Ge1—Ca1^v^	67.048 (15)	Ge1^xiv^—Ca1—Ag1^xv^	49.866 (10)
Ag1^ii^—Ge1—Ca1^v^	67.048 (15)	Mg1^xv^—Ca1—Ag1^xv^	0.000 (6)
Ag1^iii^—Ge1—Ca1^v^	66.097 (13)	Ge1—Ca1—Mg1^xvi^	137.481 (10)
Mg1^iii^—Ge1—Ca1^v^	66.097 (13)	Ge1^ii^—Ca1—Mg1^xvi^	109.433 (18)
Mg1^iv^—Ge1—Ca1^v^	70.325 (14)	Ge1^i^—Ca1—Mg1^xvi^	52.239 (12)
Ag1^iv^—Ge1—Ca1^v^	70.325 (14)	Ge1^xiii^—Ca1—Mg1^xvi^	51.633 (12)
Ca1—Ge1—Ca1^v^	135.874 (8)	Ge1^xiv^—Ca1—Mg1^xvi^	107.823 (18)
Ca1^ii^—Ge1—Ca1^v^	75.935 (6)	Mg1^xv^—Ca1—Mg1^xvi^	59.042 (16)
Ca1^i^—Ge1—Ca1^v^	137.148 (11)	Ag1^xv^—Ca1—Mg1^xvi^	59.042 (16)
Ge1^i^—Ag1—Ge1^ii^	107.58 (2)	Ge1—Ca1—Ag1^xvi^	137.481 (10)
Ge1^i^—Ag1—Ge1^vi^	108.575 (16)	Ge1^ii^—Ca1—Ag1^xvi^	109.433 (18)
Ge1^ii^—Ag1—Ge1^vi^	108.575 (16)	Ge1^i^—Ca1—Ag1^xvi^	52.239 (12)
Ge1^i^—Ag1—Ge1^vii^	115.669 (14)	Ge1^xiii^—Ca1—Ag1^xvi^	51.633 (12)
Ge1^ii^—Ag1—Ge1^vii^	115.669 (14)	Ge1^xiv^—Ca1—Ag1^xvi^	107.823 (18)
Ge1^vi^—Ag1—Ge1^vii^	100.20 (2)	Mg1^xv^—Ca1—Ag1^xvi^	59.042 (16)
Ge1^i^—Ag1—Mg1^viii^	122.12 (3)	Ag1^xv^—Ca1—Ag1^xvi^	59.042 (16)
Ge1^ii^—Ag1—Mg1^viii^	55.586 (12)	Mg1^xvi^—Ca1—Ag1^xvi^	0.00 (3)
Ge1^vi^—Ag1—Mg1^viii^	52.990 (18)	Ge1—Ca1—Mg1^xvii^	137.481 (10)
Ge1^vii^—Ag1—Mg1^viii^	121.27 (2)	Ge1^ii^—Ca1—Mg1^xvii^	52.239 (12)
Ge1^i^—Ag1—Ag1^viii^	122.12 (3)	Ge1^i^—Ca1—Mg1^xvii^	109.433 (18)
Ge1^ii^—Ag1—Ag1^viii^	55.586 (12)	Ge1^xiii^—Ca1—Mg1^xvii^	107.823 (18)
Ge1^vi^—Ag1—Ag1^viii^	52.990 (18)	Ge1^xiv^—Ca1—Mg1^xvii^	51.633 (12)
Ge1^vii^—Ag1—Ag1^viii^	121.27 (2)	Mg1^xv^—Ca1—Mg1^xvii^	59.042 (16)
Mg1^viii^—Ag1—Ag1^viii^	0.000 (16)	Ag1^xv^—Ca1—Mg1^xvii^	59.042 (16)
Ge1^i^—Ag1—Mg1^ix^	55.586 (12)	Mg1^xvi^—Ca1—Mg1^xvii^	84.104 (19)
Ge1^ii^—Ag1—Mg1^ix^	122.12 (3)	Ag1^xvi^—Ca1—Mg1^xvii^	84.104 (19)
Ge1^vi^—Ag1—Mg1^ix^	52.990 (18)	Ge1—Ca1—Ag1^xvii^	137.481 (10)
Ge1^vii^—Ag1—Mg1^ix^	121.27 (2)	Ge1^ii^—Ca1—Ag1^xvii^	52.239 (12)
Mg1^viii^—Ag1—Mg1^ix^	85.64 (3)	Ge1^i^—Ca1—Ag1^xvii^	109.433 (18)
Ag1^viii^—Ag1—Mg1^ix^	85.64 (3)	Ge1^xiii^—Ca1—Ag1^xvii^	107.823 (18)
Ge1^i^—Ag1—Ag1^ix^	55.586 (12)	Ge1^xiv^—Ca1—Ag1^xvii^	51.633 (12)
Ge1^ii^—Ag1—Ag1^ix^	122.12 (3)	Mg1^xv^—Ca1—Ag1^xvii^	59.042 (16)
Ge1^vi^—Ag1—Ag1^ix^	52.990 (18)	Ag1^xv^—Ca1—Ag1^xvii^	59.042 (16)
Ge1^vii^—Ag1—Ag1^ix^	121.27 (2)	Mg1^xvi^—Ca1—Ag1^xvii^	84.104 (19)
Mg1^viii^—Ag1—Ag1^ix^	85.64 (3)	Ag1^xvi^—Ca1—Ag1^xvii^	84.104 (19)
Ag1^viii^—Ag1—Ag1^ix^	85.64 (3)	Mg1^xvii^—Ca1—Ag1^xvii^	0.00 (2)
Mg1^ix^—Ag1—Ag1^ix^	0.000 (16)		

Symmetry codes: (i) −*x*+1, −*y*+1, −*z*+1; (ii) −*x*+1, −*y*, −*z*+1; (iii) *x*+1, *y*, *z*; (iv) *x*+1/2, *y*, −*z*+1/2; (v) −*x*+3/2, −*y*, *z*−1/2; (vi) *x*−1, *y*, *z*; (vii) *x*−1/2, *y*, −*z*+1/2; (viii) −*x*, −*y*, −*z*+1; (ix) −*x*, −*y*+1, −*z*+1; (x) *x*−1/2, *y*, −*z*+3/2; (xi) −*x*+1/2, −*y*, *z*−1/2; (xii) −*x*+1/2, −*y*+1, *z*−1/2; (xiii) −*x*+3/2, −*y*+1, *z*+1/2; (xiv) −*x*+3/2, −*y*, *z*+1/2; (xv) *x*+1/2, *y*, −*z*+3/2; (xvi) −*x*+1/2, −*y*+1, *z*+1/2; (xvii) −*x*+1/2, −*y*, *z*+1/2.
